# Three new nortriterpenoids from *Schisandra wilsoniana* and their anti-HIV-1 activities

**DOI:** 10.1007/s13659-011-0008-6

**Published:** 2011-09-02

**Authors:** Guang-Yu Yang, Yin-Ke Li, Xing-Jie Zhang, Xiao-Nian Li, Liu-Meng Yang, Yi-Ming Shi, Wei-Lie Xiao, Yong-Tang Zheng, Han-Dong Sun

**Affiliations:** 1 State Key Laboratory of Phytochemistry and Plant Resources in West China, Kunming Institute of Botany, Chinese Academy of Sciences, Kunming, 650201 Yunnan, China; 2 Key Laboratory of Tobacco Chemistry of Yunnan Province, Yunnan Academy of Tobacco Science, Kunming, 650106 Yunnan, China; 3 School of Chemistry and Biotechnology, Yunnan Nationalities University, Kunming, 650031 Yunnan, China; 4 Key Laboratory of Animal Models and Human Disease Mechanisms of the Chinese Academy of Sciences & Yunnan Province, Kunming Institute of Zoology, Chinese Academy of Sciences, Kunming, 650223 Yunnan, China

**Keywords:** nortriterpenoid, wilsonianadilactone, *Schisandra wilsoniana*

## Abstract

Three new highly oxygenated nortriterpenoids, wilsonianadilactones D–F (**1–3**), were isolated from the leaves and stems of *Schisandra wilsoniana*. Their structures were established by means of spectroscopic analysis. Compounds **1–3** showed weak anti-HIV-1 activity with the therapeutic index (TI) values (CC_50_/EC_50_) greater than 8.16, 14.7, and 17.5, respectively.

## Introduction

*Schisandra wilsoniana* A. C. Smith (Schisandraceae) is a climbing plant mainly distributed in Heqing, Lijiang, Dali, and Yulong prefectures of Yunnan Province in the mainland of China.[1,2] In previous work, a number of chemical constituents, such as nortriterpenoids,[2] carotane sesquiterpenoids,[3,4] neolignans,[5] and dibenzocyclooctadiene lignans,[6-9] were isolated from this plant. Some of these compounds showed in vitro anti-HIV-1 and anti-HBV activities.[2-9] Motivated by the discovery of new and bioactive metabolites from this plant, our group has reinvestigated the chemical constituents of the leaves and stems of *S. wilsoniana*, which led to the isolation and characterization of three new highly oxygenated nortriterpenoids, wilsonianadilactones D–F (**1**–**3**). Their structures were established by means of MS and extensive NMR spectra. This paper deals with the isolation, structural characterization, and anti-HIV-1 activity of these compounds.

## Results and Discussion

A 70% aqueous acetone extract prepared from the leaves and stems of *S. wilsoniana* was partitioned between EtOAc and $H_2O$. The EtOAc layer was subjected repeatedly to column chromatography on silica gel, Sephadex LH-20, RP-18, and semi-preparative RP-HPLC to afford compounds **1**–**3**. Their structures were shown in Figure 1, and $^1H$ and $^{13}C$ NMR spectroscopic data were listed in Tables 1 and 2.

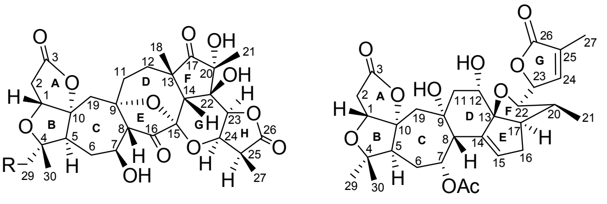

**1** R = OH
**2** R = OAc

**3**

**Figure 1** The structures of compounds **1**–**3**.

Wilsonianadilactone D (**1**) was obtained as optically active white crystals. Its molecular formula, $C_{29}H_{36}O_{13}$, was established on the basis of HRESIMS analysis ($[M + Na]^+$, *m/z* 615.2057, calcd 615.2054) and its $^1H$ and $^{13}C$ NMR spectra. Analysis of the $^1H$ and $^{13}C$ NMR (Tables 1 and 2), HSQC and HMBC (Figure 2) spectra of **1** revealed the presence of 29 carbons, including two ester carbonyl groups, two ketos, four methyls, six methylenes (including an oxygenated one), eight methines (including four oxygenated ones), and seven quaternary carbons (including six oxygenated ones), which suggested a highly oxygenated triterpene skeleton. Comparison of the1D and 2D NMR data of **1** with those of micrandilactone A[10] revealed that compound **1** was structurally similar to micrandilactone A[10]. The differences were resulted from the ap-

*To whom correspondence should be addressed. E-mail: xwl@mail.kib.ac.cn and hdsun@mail.kib.ac.cn.

pearance of an oxygenated methylene signal ($\delta_C$ 68.8 t; $\delta_H$ 3.59, 3.68, Abd, $J$=12.0 Hz), and the lack of a methyl signal ($\delta_C$ 27.7 s; $\delta_H$ 1.24 s in micrandilactone A) in **1**. This indicated that a methyl group (C-29) in micrandilactone A was replaced by an oxygenated methylene group in **1** (Figure 1). This was also

**Table1. The $^{13}$C NMR (125 MHz, Pyridine-$d_5$, $\delta$ in ppm) data of 1–3.**

| Pos. | 1 | 2 | 3 |
|---|---|---|---|
| 1 | 81.8, CH | 81.8, CH | 83.4, CH |
| 2 | 35.1, CH$_2$ | 35.1, CH$_2$ | 35.5, CH$_2$ |
| 3 | 175.2, C | 175.2, C | 175.3, C |
| 4 | 87.9, C | 85.8, C | 84.4, C |
| 5 | 55.5, CH | 56.2, CH | 54.8, CH |
| 6 | 38.0, CH$_2$ | 37.2, CH$_2$ | 34.8, CH$_2$ |
| 7 | 67.7, CH | 67.5, CH | 66.1, CH |
| 8 | 59.7, CH | 58.4, CH | 51.5, CH |
| 9 | 83.0, C | 83.2, C | 78.1, C |
| 10 | 93.9, C | 94.2, C | 98.7, C |
| 11 | 43.2, CH$_2$ | 42.2, CH$_2$ | 42.4, CH$_2$ |
| 12 | 32.5, CH$_2$ | 32.2, CH$_2$ | 74.8, CH |
| 13 | 49.3, C | 48.8, C | 98.1, C |
| 14 | 54.1, CH | 54.6, CH | 140.8, C |
| 15 | 99.8, C | 99.2, C | 130.0, CH |
| 16 | 209.9, C | 209.2, C | 31.9, CH$_2$ |
| 17 | 220.7, C | 219.9, C | 45.2, CH |
| 18 | 30.9, CH$_3$ | 30.8, CH$_3$ | |
| 19 | 41.1, CH$_2$ | 41.2, CH$_2$ | 46.5, CH$_2$ |
| 20 | 80.2, C | 79.6, C | 37.9, CH |
| 21 | 18.9, CH$_3$ | 18.5, CH$_3$ | 12.8, CH$_3$ |
| 22 | 75.5, C | 75.2, C | 82.2, CH |
| 23 | 75.2, CH | 74.6, CH | 81.4, CH |
| 24 | 76.7, CH | 75.9, CH | 147.1, CH |
| 25 | 42.5, CH | 42.2, CH | 130.7, C |
| 26 | 177.6, C | 178.1, C | 174.3, C |
| 27 | 8.1, CH$_3$ | 8.3, CH$_3$ | 10.4, CH$_3$ |
| 29 | 68.8, CH$_2$ | 70.1, CH$_2$ | 28.5, CH$_3$ |
| 30 | 16.4, CH$_3$ | 17.2, CH$_3$ | 22.3, CH$_3$ |
| OAc | | 170.3, C | 170.1, C |
| | | 21.2, CH$_3$ | 21.3, CH$_3$ |

supported by the HMBC correlations of H-29 ($\delta_H$ 3.59, 3.68) with C-4 ($\delta_C$ 87.9), C-30 ($\delta_C$ 16.4) and C-5 ($\delta_C$ 55.5); and of H-5 ($\delta_H$ 3.02) and H-30 ($\delta_H$ 1.44) with C-29 ($\delta_C$ 68.8) (Figure 2). The other chiral centers were deduced to be the same as those of micrandilactone A by analysis of ROESY spectrum of **1** (Figure 3) and comparison of chemical shifts of **1** with those of micrandilactone A.[10] Thus, the structure of **1** was established and given the name of wilsonianadilactone D.

The molecular formula of **2** was deduced as $C_{31}H_{38}O_{14}$ from its HRESIMS ([M + Na]$^+$, *m/z* 657.2166, calcd 657.2159) and its $^{13}$C NMR spectrum. The $^1$H and $^{13}$C NMR spectral data of **2** were very close to those of **1** (Tables 1 and 2). The significant chemical-shift differences observed in the NMR spectra between **1** and **2** were associated with the appearance of an additional acetoxyl group signals ($\delta_C$ 170.3 s, 21.2 q; $\delta_H$ 2.12 s) in **2**. This indicated that a hydroxyl group in **1** was replaced by an acetoxyl group in **2**. The HMBC correlation of H-29 ($\delta_H$ 3.66, 3.84) with the acetyl carbon ($\delta_C$ 170.3) suggested that the acetoxyl group should be located at C-29. Thus, the structure of **2** (Figure 1) was determined as shown and named as wilsonianadilactone E.

HRESIMS (*m/z* 581.2362, calcd 581.2363, [M + Na]$^+$) analysis of compound **3** demonstrated that it had the same

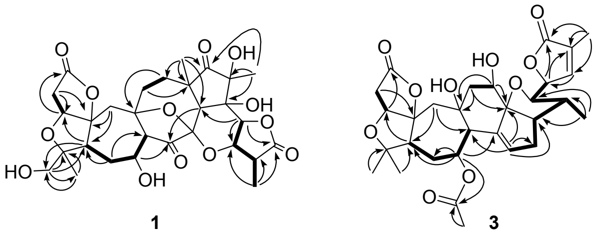

**Figure 2** Selected HMBC and $^1$H-$^1$H COSY correlations of **1** and **3**.

molecular formula of $C_{30}H_{38}O_{10}$ as that of lancifodilactone A.[11] The $^1$H and $^{13}$C NMR spectra data of **3** were very similar to those of lancifodilactone A. Analysis of HSQC, and HMBC (Figure 2) spectra of **3** showed that obvious differences were resulted from the location of acetoxyl group. The HMBC correlation of H-7 ($\delta_H$ 4.82) with the acetyl carbon ($\delta_C$ 170.1) revealed that the acetoxyl group should be located at C-7 in **3** other than at C-12 in lancifodilactone A. The ROESY correlations (Figure 3) observed in **3** and comparison of chemical shifts and coupling constants of **3** with those of lancifodilactone A[11] suggested that the two compounds have the same relative configurations. Therefore, the structure of compound **3** was determined as shown, and given the trivial name of wilsonianadilactones F (Figure 1).

Since some of nortriterpenoids from *Schisandra* spp exhibited modest or strong anti-HIV activities,[2,14-18] compounds **1–3** were tested for their potencies in preventing the cytopathic effects of HIV-1 in C8166 cells and cytotoxicity was measured in parallel with the determination of antiviral activity, using AZT as a positive control ($EC_{50}$ = 0.0045 $\mu$g/mL and $CC_{50}$ > 200 $\mu$g/mL).[19] Compounds **1–3** showed weak anti-HIV-1 ac-

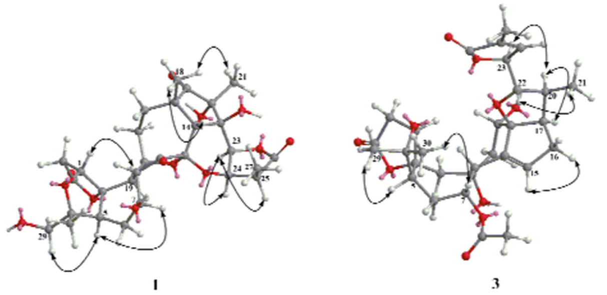

**Figure 3** Selected ROESY correlations of **1** and **3**.

tivities with $EC_{50}$ values of 24.5, 13.6 and 11.4 $\mu$g/mL, respectively, and all exerted minimal cytotoxicity against C8166 cells ($CC_{50}$ > 200 $\mu$g /mL). The therapeutic index (TI) values ($CC_{50}/EC_{50}$) of **1–3** were greater than 8.16, 14.7, and 17.5, respectively.

**Table 2. The $^1$H NMR (500 MHz, Pyridine-$d_5$, $\delta$ in ppm, $J$ in Hz) data of 1–3.**

| Pos. | 1 | 2 | 3 | Pos. | 1 | 2 | 3 |
|---|---|---|---|---|---|---|---|
| 1 | 4.17, d (6.2) | 4.27, d (5.9) | 4.18, d (4.6) | 17 | | | 2.99, m |
| 2α | 2.94, dd (6.4, 18.5) | 2.89, dd (6.2, 18.0) | 2.99, dd (4.5, 18.2) | 18 | 1.57, s | 1.58, s | |
| 2β | 2.75, d (18.5) | 2.71, d (18.5) | 2.65, d (17.4) | 19α | 2.24, ABd (15.5) | 2.50, ABd (15.3) | 2.02–2.07, overlap |
| 5 | 3.02, dd (4.5,13.5) | 2.99, dd (4.5, 13.1) | 3.29, dd (4.6, 13.0) | 19β | 2.52, ABd (15.5) | 2.21, ABd (15.3) | 1.95, ABd (15.8) |
| 6α | 1.95–1.98, overlap | 1.93–1.97, overlap | 2.02–2.07, overlap | 20 | | | 2.38, m |
| 6β | 2.22–2.47, m | 2.23-2.48, m | 1.54–1.59, m | 21 | 1.60, s | 1.58, s | 0.93, d (6.6) |
| 7 | 4.40, dd (9.2, 10.4) | 4.38, dd (9.2, 10.2) | 4.82, br s | 22 | | | 3.68, dd (4.3, 9.6) |
| 8 | 2.99, d (10.4) | 2.96, d (10.2) | 2.60, br s | 23 | 4.96, d (1.8) | 4.93, d (2.1) | 5.12, br s |
| 11α | 1.95–1.98, overlap | 1.93–1.97, overlap | 2.28, dd (2.6, 15.4) | 24 | 5.40, dd (1.8, 2.2) | 5.42, dd (1.8, 2.5) | 7.28, br s |
| 11β | 1.74–1.79, m | 1.72–1.76, m | 2.16, dd (2.6, 15.4) | 25 | 3.23–3.27, m | 3.20–3.25, m | |
| 12α | 1.95–1.98, overlap | 1.93–1.97, overlap | 5.26, br s | 27 | 1.20, d (7.1) | 1.22, d (7.2) | 1.82, s |
| 12β | 1.58–1.62, m | 1.55–1.59, m | | 29α | 3.59, Abd (12.0) | 3.66, ABd (12.1) | 1.98, s |
| 14 | 3.23, s | 3.20, s | | 29β | 3.68, Abd (12.0) | 3.84, ABd (12.1) | |
| 15 | | | 6.27, br s | 30 | 1.44, s | 1.46, s | 0.96, s |
| 16 | | | 2.35, m | OAc | | 2.12, s | 2.03, s |

## Experimental Section

**General Experimental Procedures.** Optical rotations were measured with a Horiba SEPA-300 polarimeter. UV spectra were obtained using a Shimadzu UV-2401A spectrophotometer. A Tenor 27 spectrophotometer was used for scanning IR spectroscopy with KBr pellets. 1D and 2D NMR spectra were recorded on DRX-500 spectrometers. Unless otherwise specified, chemical shift ($\delta$) were expressed in ppm with reference to the solvent signals. Mass spectra were performed on a VG Autospec-3000 spectrometer. Column chromatography was performed with silica gel (200–300 mesh, Qing-dao Marine Chemical, Inc., Qingdao, China). Preparative HPLC was performed on an Agilent 1100 liquid chromatograph with a Zorbax SB-$C_{18}$ column (9.4 mm × 25 cm). Fractions were monitored by TLC and spots were visualized by heating silica gel plates sprayed with 10% $H_2SO_4$ in EtOH.

**Plant Materials.** The leaves and stems of *S. wilsoniana* were collected in Heqing prefecture of Yunnan Province, China, in August 2006. The specimen was identified by Prof. Xi-Wen Li and a voucher specimen (No. KIB 2008-08-12) has been deposited at the State Key Laboratory of Phytochemistry and Plant Resources in West China, Kunming Institute of Botany, Chinese Academy of Sciences.

**Extraction and Isolation.** The dried leaves and stems of *S. wilsoniana* (5.0 kg) were powdered and extracted with 70% aqueous $Me_2CO$ (8 L × 4) for 24 hours at room temperature and filtrated. The filtrate was concentrated and partitioned with EtOAc. The EtOAc part (83.6 g) was chromatographed on silica gel column eluting with $CHCl_3$-MeOH (1:0, 9:1, 8:2, 2:1, 1:1, and 0:1) to afford fractions I-VI. Fraction II (9:1, 14.4 g) was repeatedly chromatographed on silica gel (200–300 mesh) and Sephadex LH-20, then purified by preparative HPLC ($MeOH:H_2O$, 45:55 and 40:60, and $MEOH:CH_3CN:H_2O$, 10:40:50 and 15:35:50) to yield compounds **1** (11.5 mg), **2** (8.3 mg) and **3** (14.7 mg).

**WilSonianadilactone D** (**1**): colorless prisms; mp 180–181 °C; $[\alpha]_D^{20}$ + 46.8 (*c* 0.25, MeOH); UV: end absorption; IR (KBr) $\nu_{max}$ 3442, 2978, 2936, 1778, 1738, 1636, 1452, 1374, 1228, 1211, 1121, 1101, 1062, 1040, 1002, 915, 865 cm$^{-1}$; $^1$H and $^{13}$C NMR, data see Tables 1 and 2; positive ion ESIMS *m/z* 615 [M + Na]$^+$; HRESIMS *m/z* 615.2057 (calcd for $C_{29}H_{36}NaO_{13}$ [M + Na]$^+$, 615.2054).

**Wilsonianadilactone E** (**2**): colorless crystals; mp 184–185 °C; $[\alpha]_D^{20}$ + 43.2 (*c* 0.25, MeOH); UV: end absorption; IR (KBr) $\nu_{max}$ 3448, 2962, 2925, 1792, 1743, 1735, 1654, 1460, 1371, 1346, 1116, 1012 cm$^{-1}$; $^1$H and $^{13}$C NMR data see Tables 1 and 2; positive ion ESIMS *m/z* 657 [M + Na]$^+$; HRESIMS *m/z* 657.2166 (calcd for $C_{31}H_{38}NaO_{14}$ [M + Na]$^+$, 657.2159).

**Wilsonianadilactone F** (**3**): colorless crystals; mp 195–196 °C; $[\alpha]_D^{20}$ + 25.8 (*c* 0.24, MeOH); UV: end absorption; IR (KBr) $\nu_{max}$ 3443, 2972, 2935, 1762, 1729, 1628, 1634, 1456, 1376, 1248, 1186, 1067, 1028, 1008 cm$^{-1}$; $^1$H and $^{13}$C NMR data see Tables 1 and 2; positive ion ESIMS *m/z* 581 [M + Na]$^+$; HRESIMS *m/z* 581.2362 (calcd for $C_{30}H_{38}NaO_{10}$ [M + Na]$^+$, 581.2363).

**Cytotoxicity Assay.** The cytotoxicity assay against C8166 cells ($CC_{50}$) was assessed using the MTT method and anti-HIV-1 activity was evaluated by the inhibition assay for the cytopathic effects of HIV-1 ($EC_{50}$).[19]

## Acknowledgments

This project was supported financially by the NSFC (No. 20802082 and 30830115), the projects from the Chinese Academy of Sciences (KSCX2-EW-Q-10, KSCX1-YW-R-24 and KSCX2-YW-R-185), the Major State Basic Research Development Program of China (No. 2009CB522303 and 2009CB940900), the Yong Academic and Technical Leader Rising Foundation of Yunnan Province (2006PY01-47), and the Natural Science Foundation of Yunnan Province (2005XY04 and 2006B0042Q).

**Open Access** This article is distributed under the terms of the Creative Commons Attribution License which permits any use, distribution, and reproduction in any medium, provided the original author(s) and source are credited.
